# Effects of time-restricted feeding on letrozole-induced mouse model of polycystic ovary syndrome

**DOI:** 10.1038/s41598-023-28260-5

**Published:** 2023-02-02

**Authors:** Ki-Jin Ryu, Hyuntae Park, Young In Han, Hee Jung Lee, Seunghyun Nam, Hye Gyeong Jeong, Tak Kim

**Affiliations:** Department of Obstetrics and Gynecology, Korea University College of Medicine, 73 Goryeodae-Ro, Seongbuk-Gu, Seoul, 02841 Republic of Korea

**Keywords:** Endocrine reproductive disorders, Obesity, Weight management

## Abstract

The present study aimed to investigate whether time-restricted feeding (TRF) ameliorates metabolic and reproductive phenotypes in a letrozole-induced mouse model of polycystic ovary syndrome (PCOS). Sixty female C57BL/6 N mice were randomly divided into two groups according to the type of food received: either a chow or a 60% high-fat diet. Those mice were subcutaneously implanted with letrozole or placebo pellets at four weeks of age. Then, letrozole-treated mice were randomly assigned to different feeding regimens: (1) TRF for 4 h (ZT12–ZT16) or (2) ad libitum diet. After 4 weeks of dietary intervention, estrous cycles were determined with daily vaginal smear examination, and serial tail-tip blood sampling was performed at 5-min intervals for 2 h to measure the luteinizing hormone (LH) pulse frequency, amplitude, and mean LH levels in the diestrus cycle stage. Letrozole-treated mice in the ad libitum group demonstrated multiple PCOS-like phenotypes including ovulatory dysfunction, polycystic ovaries, and increased body weight, parametrial fat weight, adipocyte size and inflammation, and higher expression of *Cyp17*, *Cyp19*, and *Fshr* in the ovary, and *Kiss1r* and *Gnrh* in the hypothalamus, elevated serum testosterone levels, and more rapid and elevated LH pulsatility, with increased pulse frequency, amplitude, and mean levels in the diestrus stage, compared with the controls. After TRF for 4 weeks, those phenotypes reverted to normal levels in letrozole-treated mice, except the percentage of diestrus cycles indicating the arrest of estrous cycling which did not differ between the TRF and ad libitum groups. Our results demonstrate that TRF has therapeutic effects on the reproductive and metabolic phenotypes of a letrozole-induced mouse model of PCOS.

## Introduction

Polycystic ovary syndrome (PCOS) is the most frequent hormonal and metabolic disorder in women of reproductive age and is characterized by hyperandrogenemia, menstrual dysfunction, and polycystic ovaries^1^. PCOS is associated with various comorbidities, including infertility, obesity, and type 2 diabetes^2–5^. Concerning PCOS etiology, an increase in the frequency and amplitude of pulsatile gonadotropin-releasing hormone (GnRH) release from hypothalamus, which in turn stimulates luteinizing hormone (LH) secretion from the pituitary gland, is known to induce hyperandrogenism and arrest follicle development in the pre-ovulatory stage^6,7^. Kisspeptin-neurokinin B-dynorphin (*KNDy*) neurons in the arcuate nucleus of the hypothalamus have been accepted as GnRH pulse generators, thus playing a major role in the pathophysiology of PCOS^8,9^. PCOS underlying mechanisms are not fully understood; therefore, research studies using PCOS animal models are crucial for exploring its in vivo pathophysiology^10^.

No therapeutic treatments for PCOS have been implemented so far, and recent guidelines have emphasized the importance of lifestyle modifications in the management of PCOS. Healthy eating and regular physical activity are strongly recommended to maintain a healthy weight and optimize hormonal outcomes^1^. However, these guidelines do not specify any dietary type or regimen. Furthermore, it is uncertain whether lifestyle modifications emphasizing weight reduction are effective in women with PCOS who are not overweight, such as in East Asian women^11^. Time-restricted feeding (TRF) is a dietary approach that involves shortening the duration of the daily eating windows without necessarily altering the diet quality and caloric intake^12^. TRF appears as a more feasible alternative for women attempting diet modifications, as calorie restriction is a major stressor in these cases^18^. Growing evidence indicates that TRF has therapeutic effects on various metabolic and inflammatory disorders^13–17^. The effects of TRF on overweight women with PCOS have been investigated. Beneficial effects, including reduced body fat, regular menstrual periods and flow, and alleviation of both hyperandrogenemia and chronic inflammation, were reported, although the study was limited by the small number of participants and inability to assess the underlying physiological mechanism^19^. Further studies are warranted to confirm these findings and elucidate the underlying mechanisms.

We aimed to investigate whether TRF regimen has beneficial effects on reproductive and metabolic manifestations in an animal model of PCOS induced by letrozole, a nonsteroidal aromatase inhibitor. We investigated whether pulsatile secretion of LH was altered following TRF treatment, along with sex hormone levels, ovulatory activity, and metabolic phenotypes.

## Materials and methods

### Animals and treatment

**Figure 1:**
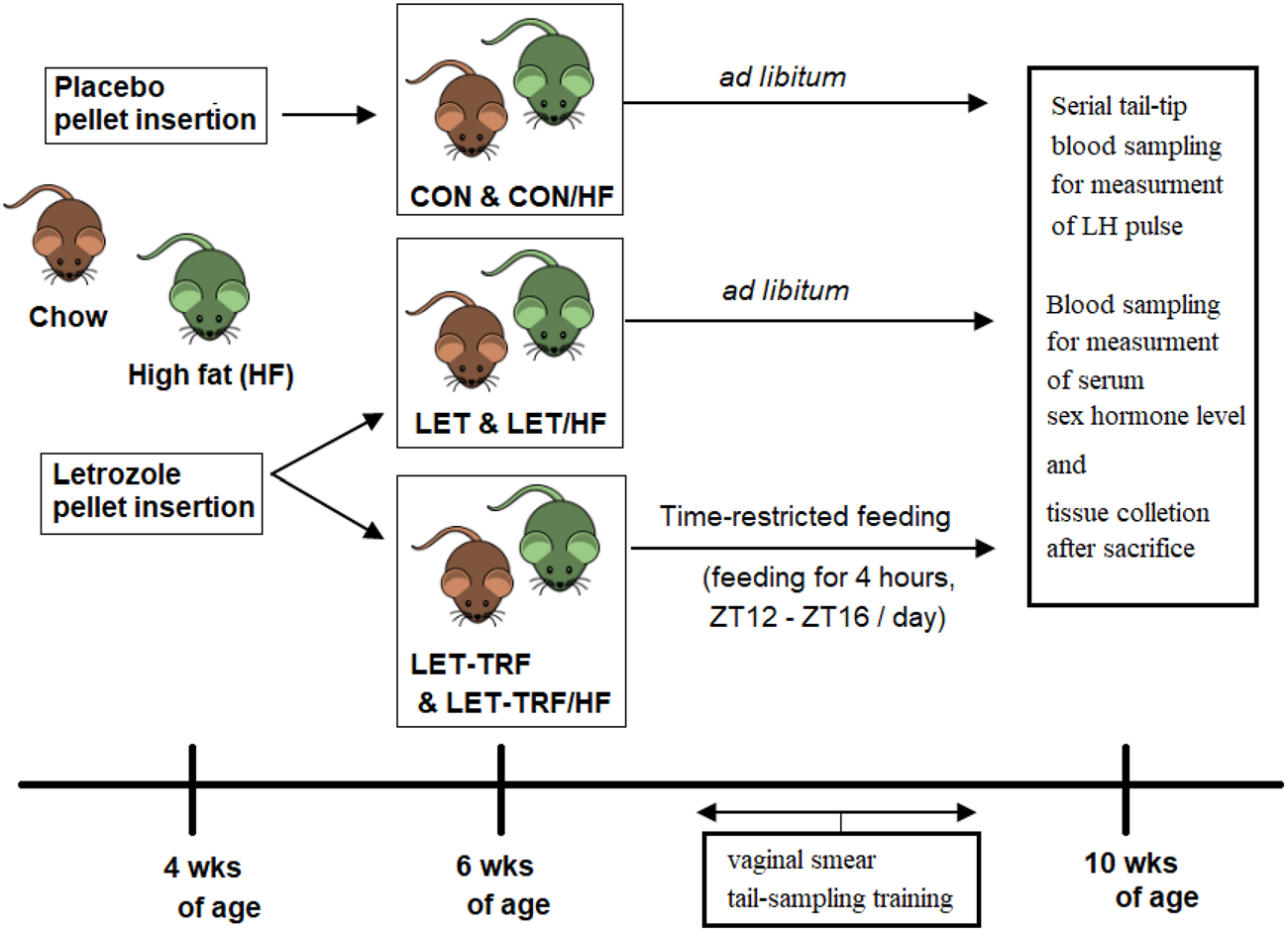
Experimental design of the study. C57BL/6 female mice were implanted with pellets containing letrozole or placebo at four weeks of age. At six weeks of age, half of the letrozole-treated mice continued ad libitum, while the other half started time-restricted feeding. Consequently, three groups were created: CON, LET, and LET-TRF. Body weights were measured twice per week throughout the study period. Vaginal smears were taken from 8 weeks of age to 10 weeks of age to assess the estrous cycle stage. Serial tail-tip blood sampling was performed to measure LH pulse at 10 weeks of age after 2–3 weeks of training for those procedures. The mice were euthanized at the end of the experiment, and tissues and serum were collected for analysis. All experiments were divided into CHOW diet and 60% HFD groups according to the type of diet fed to the mice. *CON* control, *LET* letrozole-treated, *HF*, high-fat diet, *wk* weeks.

Sixty female C57BL/6 N mice (Orient Bio, Seoul, South Korea) were housed from 3 weeks of age, with a 12:12 light–dark cycle and food and water ad libitum. Mice were randomly divided into two groups (*n* = 30 each) according to the type of food received: either a chow (CHOW) or a 60% high-fat (HF) diet. CHOW mice were in turn divided into three groups as follows: (1) LET group comprising letrozole-treated mice with food available ad libitum (*n* = 10), (2) CON group comprising placebo-treated mice with food available ad libitum (*n* = 10), and (3) LET-TRF group comprising letrozole-treated mice treated with TRF from 6 to 10 weeks of age (*n* = 10). Consistently, mice fed with HFD were also divided into three groups as follows: LET/HF, CON/HF, and LET-TRF/HF (Fig. 1). The study protocol was approved by the Institutional Animal Care and Use Committee of the Korea University College of Medicine Laboratory Animal Research Center (KOREA-2020–0003). All methods were performed in accordance with the relevant guidelines and regulations. This study was conducted in accordance with ARRIVE guidelines (https://arriveguidelines.org).

At 4 weeks of age, mice were subcutaneously implanted with a letrozole (50 µg/day) pellet (LET, LET-TRF, LET/HF, and LET-TRF/HF mice) or a placebo control pellet (CON and CON/HF mice). Letrozole powder was purchased from Fitzgerald (Acton, MA, USA) and custom 60-day continuous-release pellets were obtained from Innovative Research of America (Sarasota, FL, USA). The LET, LET/HF, CON, and CON/HF mice were housed with food and water available ad libitum; however, both LET-TRF and LET-TRF/HF mice were treated with TRF after two weeks of letrozole pellet implantation. The TRF regimen allowed the mice to eat a regular diet for 4 h (ZT12–ZT16) and fast for the remaining 20 h. Water was freely available under all conditions. No caloric restrictions were imposed during the feeding period. The amount of food intake was measured daily, and the mice body weight was tracked twice per week from 4 to 10 weeks of age.

### Estrous cycle assessment

Vaginal cytology of females was examined daily at approximately 09:00 AM to determine the stage of the estrous cycle, which was determined by microscopic visualization of the predominant cell type in vaginal epithelial smears obtained on that same day, at 4–5 weeks after LET or control pellet implantation over a 10-day period^20^. The percentage of diestrus cycles over 10 days was compared among the three groups.

### Serial tail-tip blood sampling for the measurement of LH pulse

Serial tail-tip blood sampling of mice was conducted to assess the pattern of pulsatile LH secretion as previously published^21^ with slight modifications. Mice were subjected to daily handling for 5–10 min from 7 weeks of age (3 weeks after LET pellet implantation) for habituation. From 10 weeks of age (6 weeks after pellet implantation), whenever mice were confirmed to be in diestrus stage, serial sampling was conducted between 9:00 AM and 12:00 PM. Briefly, the tail tip was cut, and approximately 15 min later, serial blood samples were collected every 5 min for a total duration of 2 h. For each sample, 3 μL of whole blood was pipetted from the tail, mixed with 57 μL of assay buffer (0.2% Bovine Serum Albumin—0.05% Tween 20—PBS, pH 7.5), and placed on ice until being stored at − 20 °C. The animals were awake for the entire duration of the experiment and able to freely roam in their home cages between sampling sessions. LH levels were measured using an ultrasensitive mouse and rat LH enzyme-linked immunosorbent assay (ELISA) from the University of Virginia Center for Research in Reproduction Ligand Assay and Analysis Core.

### LH pulse identification and analysis

Endogenous LH pulse peaks were identified according to DynPeak algorithm^21–23^. Briefly, a pulse peak was defined by the following criteria: (1) LH peak value increases by > 20% compared to the previous one or two LH values; (2) LH peak value is followed by a decrease of > 10% in the subsequent one or two LH values; and (3) the change in pulse amplitude is ≥ 0.320 ng/mL (sensitivity of the assay)^24^. In addition to the pulse peaks, the following parameters were calculated for LH pulsatility: (1) pulse frequency per 60 min; (2) pulse amplitude, defined as the difference between the pulse peak value and a preceding nadir, the lowest value of the three preceding values; and (3) overall mean LH level, defined as the average of all LH values for an animal for the entire 2 h sampling period^22^.

### Tissue collection and histology

After seven weeks of letrozole exposure, mice were anesthetized with isoflurane and weighed, blood samples were collected via cardiac puncture, and mice were euthanized by CO_2_ inhalation. Both ovaries and parametrial fat pads were dissected. One ovary and parametrial fat pad from each mouse were fixed overnight in 4% paraformaldehyde at 4 °C and stored in 70% ethanol prior to histological processing. The other ovary and fat pad were dissected and stored in RNAlater (Life Technologies, NY, USA) at − 80 °C until tissue processing for mRNA extraction and quantification via reverse transcription-polymerase chain reaction (RT-PCR). Frozen brains were dissected, and micropunches were taken from the anterior hypothalamus/preoptic area (POA).

For histological analysis, fixed ovaries were embedded in paraffin and serial 12-μm-thick sections were obtained and stained with hematoxylin and eosin. The number of cystic follicles was counted in two areas randomly selected from the middle ovary region. Fixed parametrial fat pads were embedded in paraffin, sectioned into 5-μm slices, and stained with hematoxylin and eosin. Slides were scanned using a Hamamatsu Nanozoomer, and adipocyte numbers and areas were quantified using ImageJ. Macrophage infiltration in additional adipose tissue sections was assessed by immunohistochemistry using the F4/80 antibody (1:100, clone CI:A3-1; Serotec, Oxford) with 3,3’-diaminobenzidine detection.

### Serum sex hormone levels

Cardiac puncture blood samples were collected at the end of the study to analyze serum testosterone (T) and estradiol (E2) levels, when mice were confirmed to be in diestrus stage. Serum was collected and stored at − 20 °C. Hormone levels were measured at the University of Virginia Ligand Core Facility. Serum T levels were measured using radioimmunoassay (range: 5.0–1075 ng/dL). Serum E2 levels were measured using a mouse enzyme-linked immunosorbent assay (range: 3.0–300 pg/mL). For each hormone assay, all measurements were above the detection limit of the assay.

### Quantitative real-time PCR of ovary, parametrial fat, and hypothalamus tissues

Total RNA from the ovary, parametrial fat, and POA samples was isolated using an RNeasy Mini kit (Qiagen, Hilden, Germany). Genomic DNA was eliminated using the Turbo DNA-free kit (Ambion). Reverse transcription was performed using an iScript cDNA synthesis kit (Bio-Rad Laboratories, Hercules, CA, USA). Complementary DNA products were detected using the iQ SYBR Green Supermix (Bio-Rad Laboratories) on a quantitative real-time PCR iQ5 real-time detection system (Bio-Rad Laboratories). Data were analyzed by the 2^−△△Ct^ method by normalizing the ovariangene of interest to L19 and pituitary or adipose genes of interest to Gapdh. Data are represented as mean fold change compared with control ± SEM.Primer sequences used are listed in Supplementary Table 1.

### Statistical analysis

Data are shown as the mean ± standard error of the mean, unless otherwise stated. Statistical analyses were performed using unpaired two-tailed Student’s *t*-test or two-way analysis of variance (ANOVA). For all analyses, statistical significance was set at* P* < 0.05, and statistical analyses were performed using SPSS software (version 20.0; IBM Corp., Armonk, NY, USA).

### Attestation statements

Data regarding any of the subjects in the study has not been previously published unless specified. Data will be made available to the editors of the journal for review or query upon request.

### Ethical approval

All experiments were conducted according to the guidelines of the ARRIVE, and approved by the Institutional Animal Care and Use Committee of the Korea University College of Medicine Laboratory Animal Research Center (KOREA-2020–0003).

## Results

### Pulsatile secretion patterns of LH at diestrus and serum sex hormone levels

**Figure 2:**
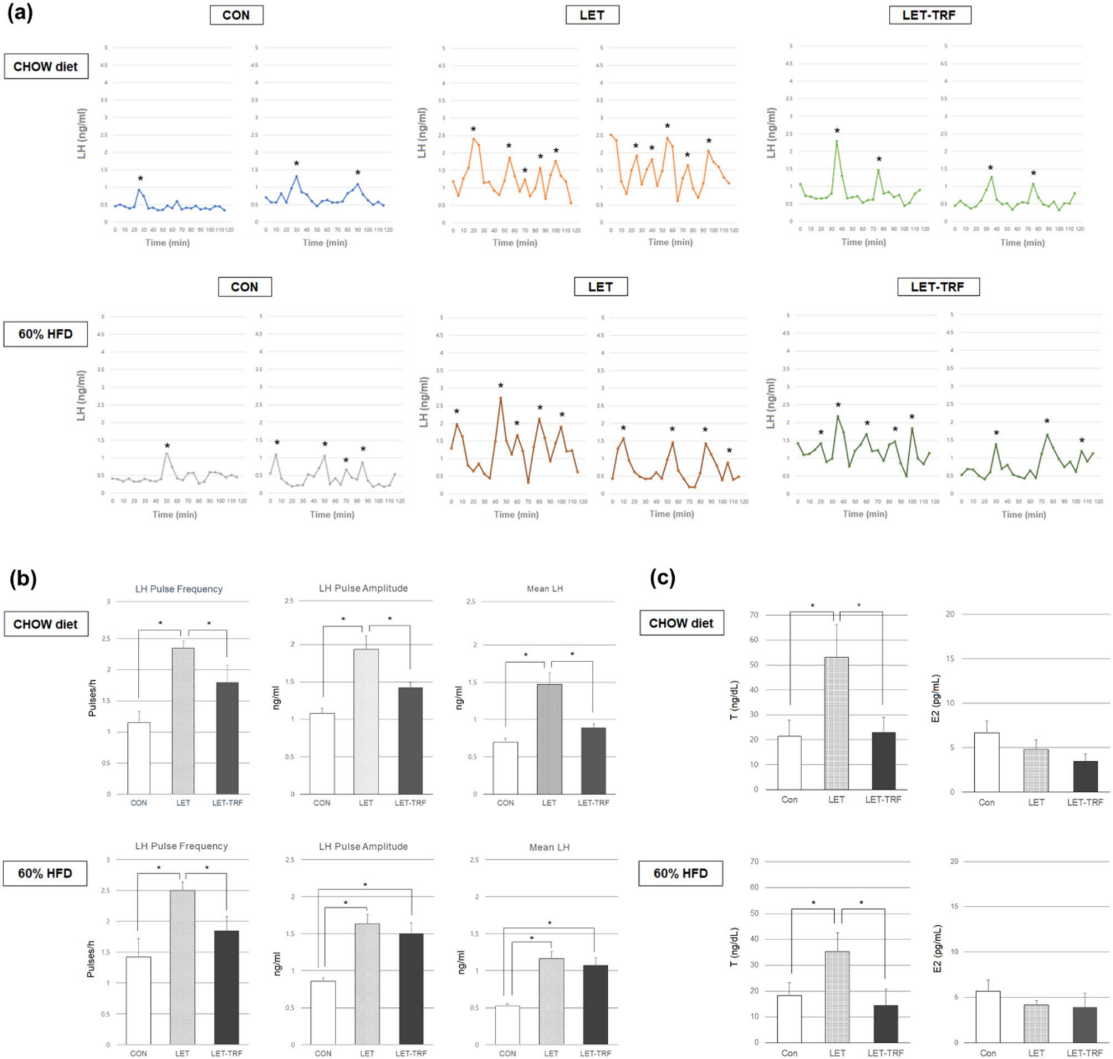
Endogenous pulsatile LH secretion profiles and serum sex hormone levels. (**a**) Representative LH pulse profiles from each of the two mice at diestrus in the CON, LET, and LET-TRF groups, which were fed either a CHOW diet or 60% HFD. Blood was collected every 5 min for a total duration of 2 h to generate LH pulse profiles at 10 weeks of age (6 weeks after letrozole or placebo pellet implantation). LET-TRF mice were fed the TRF regimen for 4 weeks, whereas the other mice were fed ad libitum. The asterisks indicate the identified pulse peaks. (**b**) Quantitative analyses of endogenous pulsatile LH secretion parameters. LH pulse parameters, including pulse frequency, pulse amplitude, and mean LH levels, were compared between the study groups (^*^P < 0.05). (**c**) Comparison of mean serum levels of T and E2 between the study groups (^*^P < 0.05). Serum was obtained at 10 weeks of age (6 weeks after letrozole or placebo pellet implantation). LET-TRF mice were fed the TRF regimen for 4 weeks, whereas the other mice were fed ad libitum. *LH* luteinizing hormone; 60% HFD, 60% kcal high-fat diet; *TRF* time-restricted feeding, *T* testosterone, *E2* estradiol.

LET mice, both on CHOW and 60% HFD, exhibited a more hyperactive pulsatile LH secretion pattern, along with a higher pulse frequency, amplitude of each pulse, and mean LH levels than CON mice (Fig. 2a, b). However, LET-TRF mice demonstrated a pulsatile LH secretion pattern similar to that of CON mice.

LET mice had increased T levels, mirroring hyperandrogenemia in women with PCOS, whereas LET-TRF mice did not (Fig. 2c). In contrast, circulating E2 levels were not different among the three groups. Altered serum T levels in LET mice were also observed in the 60% HFD treatment, although the shift was smaller than that observed with the CHOW treatment (Fig. 2c).

### Body weight changes, and parametrial fat adiposity and inflammation

**Figure 3:**
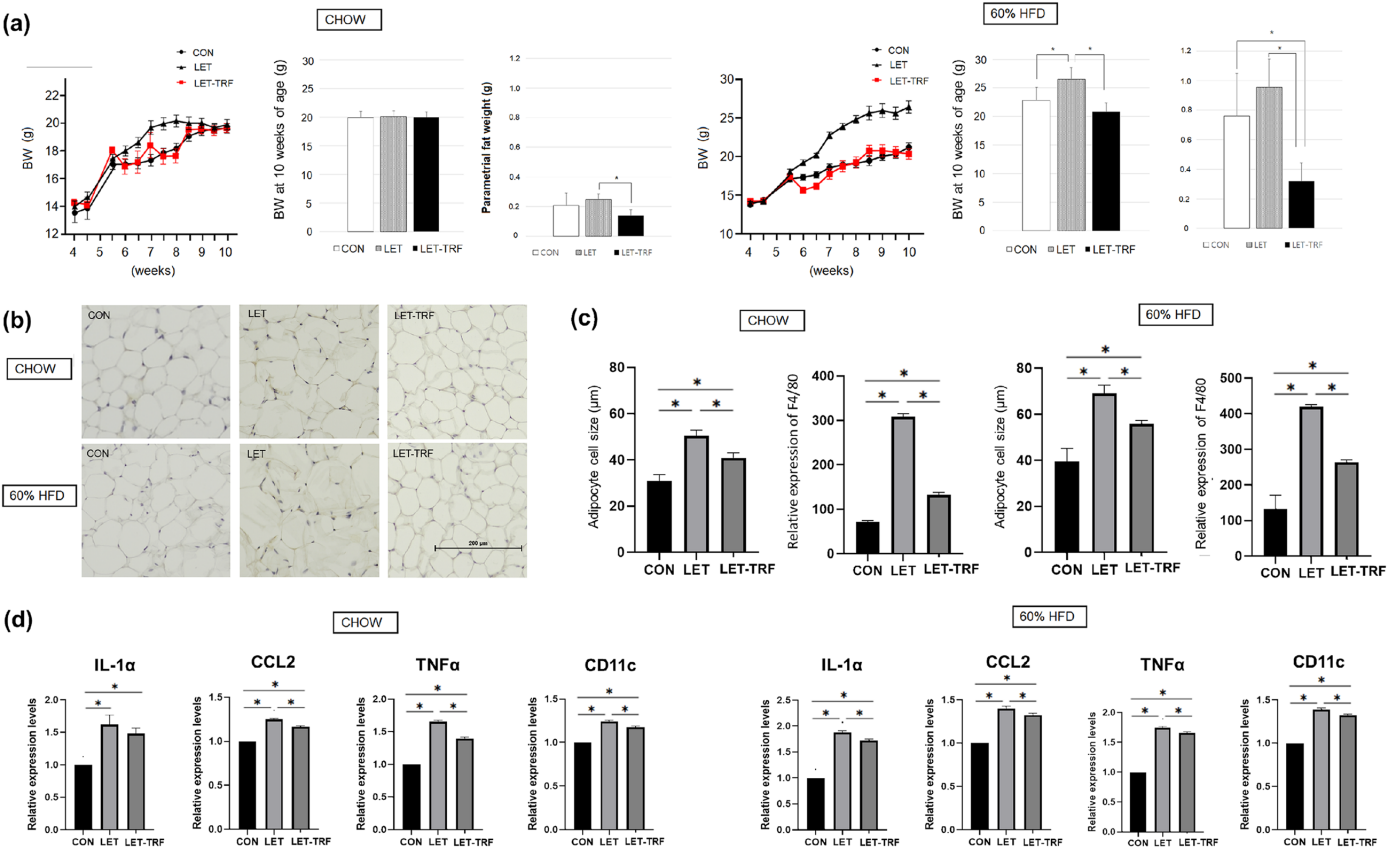
Body weight, adiposity and inflammation markers in parametrial fat tissues. (**a**) Linear graphs present the body weight change of mice in the study groups, divided by the type of diet, during the 6-week study period. The bar graphs present the mean comparison of body weights measured at 10 weeks of age and the weight of parametrial fat harvested from mice after they were sacrificed at 10 weeks of age. (**b**) Representative pictures of F4/80-stained adipose cells of parametrial fat pads are presented. (**c**) Comparison of the mean adipocyte size and relative expression of F4/80-stained cells in parametrial fat tissues according to the study groups. (**d**) Relative expression levels of inflammatory markers in adipocyte tissue, including IL-1α, CCL2, TNF-α, and CD11c, were compared. ^*^P < 0.05. 60% HFD, 60% kcal high-fat diet, *TRF* time-restricted feeding.

Among mice fed the CHOW diet, LET mice showed a more rapid initial weight gain from 6 to 8 weeks of age. However, the rate of weight gain gradually decreased thereafter to reach levels to those observed with CON mice (Fig. 3a). In contrast, among 60% HFD-treated mice, LET mice displayed a steady increase in body weight compared with CON and LET-TRF mice, and showed a significantly higher body weight at 10 weeks of age (Fig. 3a).

The weight of the parametrial fat pad was significantly lower in LET-TRF mice than in LET mice (CHOW treatment) or in both LET and CON mice (60% HFD treatment) (Fig. 3a). The average adipocyte size of the LET mice was higher than that of CON and LET-TRF mice in both the CHOW and 60% HFD treatments (Fig. 3b). Macrophage infiltration in adipose tissue sections appeared with higher frequency in LET mice than in CON mice. LET-TRF mice also showed more frequent macrophage infiltration in adipose tissue than CON mice; however, it was significantly less frequent compared to that in LET mice (Fig. 3b, c). Relative expression levels of inflammatory markers in adipocyte tissues, including interleukin 1 alpha (IL-1α), C–C Motif Chemokine Ligand 2 (CCL2), tumor necrosis factor alpha (TNF-α), and CD11c, were higher in LET and LET-TRF mice than in CON mice. However, most of these markers showed lower levels in LET-TRF mice compared to LET mice (Fig. 3d).

### Histologic findings of ovary and the assessment of estrous cycling

**Figure 4:**
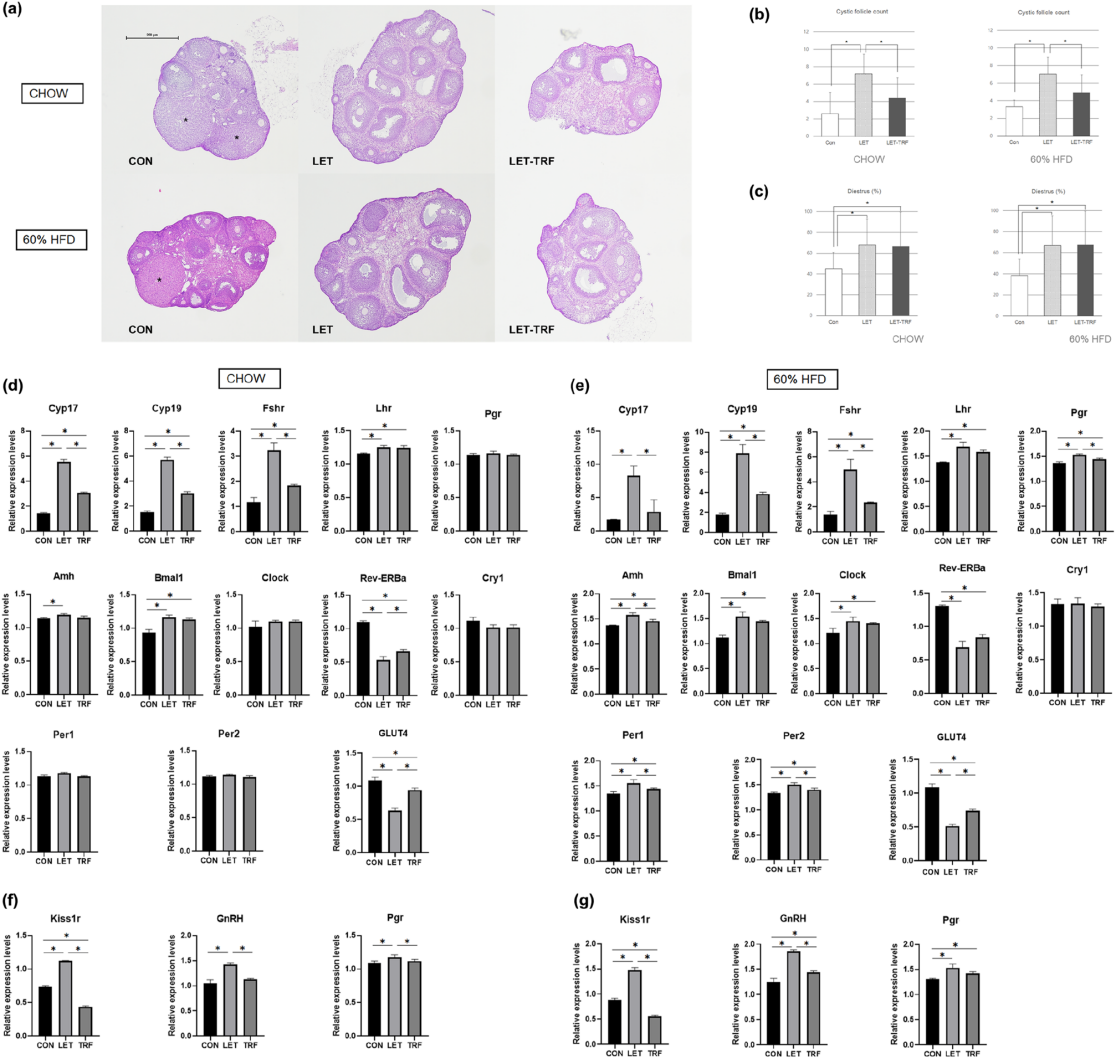
Histological findings of the ovaries, assessment of ovulatory disfunction, and the ovary/hypothalamus expression of key reproductive or circadian rhythm-associated genes. (**a**) Representative images of histological findings of ovaries with H&E staining from CON, LET, and LET-TRF mice according to diet. The ovaries of LET mice showed virtually enlarged and polycystic morphologies compared to those of CON and LET-TRF mice, either in CHOW or 60% HFD. Asterisks indicate the identified corpus luteal cysts. (**b**) Mean analyses of cystic follicle count and (**c**) Percentage of diestrus cycle in CHOW and 60% HFD treatments. (**d**) Mean expression levels of mRNAs in the ovaries from the CHOW diet treatment and (**e**) those from HFD experiment. (**f**) Mean expression levels of mRNAs in the anterior hypothalamus/POA samples from the CHOW diet treatment and (**g**) those from HFD treatment. ^*^P < 0.05. 60% HFD, 60% kcal high-fat diet, *TRF* time-restricted feeding.

The ovaries of LET mice showed an enlarged polycystic morphology and a higher number of cystic follicles, consistent with a PCOS phenotype (Fig. 4a, b). However, the ovaries of LET-TRF mice showed a morphology similar to those of CON mice. Both LET and LET-TRF mice showed a higher percentage of diestrus stage, indicating a higher probability of estrous cycle arrest in these mice (Fig. 4c). There was no significant difference in diestrus percentage between LET and LET-TRF mice.

### Comparison of expression of messenger RNAs in the ovary and hypothalamus

LET mice showed higher expression of reproductive state-associated mRNAs, including *Cyp17*, *Cyp19*, *Fshr, Lhr,* and *Amh* in the ovary (Fig. 4d). Among them, *Cyp17*, *Cyp19,* and *Fshr* levels were markedly decreased in the ovaries of LET-TRF mice, although they did not reach the levels observed in CON mice. Among the six circadian rhythm-associated mRNAs, *Rev-erbα* decreased markedly in LET mice compared to CON mice, and slightly increased again in TRF mice. *Bmal1* expression was higher in LET; however, there was no difference between LET and LET-TRF mice (Fig. 4d). Furthermore, the expression of *GLUT-4*, a marker for insulin responsiveness decreased in LET mice compared to that in CON and LET-TRF mice. The three reproductive mRNAs in the anterior hypothalamus/POA samples, *Kiss1r, Gnrh,* and *Pgr,* were increased in LET mice; however, all showed decreased levels in LET-TRF mice (Fig. 4f). The results from the HFD treatment showed trends similar to the abovementioned results from the CHOW treatment (Fig. 4e, g).

## Discussion

This study showed that TRF had a positive effect on both reproductive and metabolic phenotypes in a LET-induced PCOS mouse model. In line with the previous studies, when compared with CON mice, LET mice showed several phenotypes of PCOS, including higher serum T and LH levels, ovulatory dysfunction, polycystic ovary morphology, higher body weight, higher adipocyte size and inflammation. However, most of these phenotypes were restored after 4 weeks of TRF (LET-TRF mice): lower T, LH, cystic follicle count in the ovary, body weight (in the HFD diet experiment), parametrial fat weight, adipocyte size, and inflammation, than the values observed in mice that received the ad libitum diet (LET mice). In particular, LET mice showed a remarkably hyperactive pulsatile secretion pattern of LH, whereas LET-TRF mice showed a normal pattern of LH secretion, similar to the pattern of LH secretion exhibited by CON mice. TRF was also associated with low expression of *Cyp17*, *Cyp19*, and *Fshr* in the ovary, and low expression of *Kiss1r* and *Gnrh* in the hypothalamus compared with the ad libitum diet in LET mice. These results indicate that TRF is a promising dietary intervention for the management of PCOS. However, ovulatory dysfunction induced in LET mice, which caused a marked increase in the percentage of diestrus cycle, was not recovered in LET-TRF mice, despite the aforementioned recoveries of sex hormone profiles and metabolic variables after TRF for 4 weeks in these mice. Further studies are warranted to test whether more effective TRF strategies can induce complete recovery of reproductive functions in patients with PCOS. Various TRF regimens regarding time division (*e.g.*, number of hours of access to food during the active phase), total duration, or starting point of TRF (*e.g.*, early pubertal age or adult period) may affect the outcomes of TRF in PCOS models.

Kauffman et al. developed a mouse model of PCOS which recapitulated the phenotypes associated with human PCOS^21,25^. In the present study, using this LET-induced mouse model of PCOS, we reconfirmed that several phenotypes were present in our model; however, others were not. In particular, mean LH levels of LET mice were much lower than those observed in previous reports^21,25^, although more hyperactive secretion of LH than in CON mice was also observed in this study. The body weight of LET mice 4 weeks after pellet insertion was comparable to that of CON mice fed the CHOW diet, unlike results from previous studies in which the body weight of LET mice was steadily higher^21,25,26^. The reason for these discrepancies is not clear. Further studies are required to confirm these results.

The beneficial effects of TRF on metabolic health profiles were confirmed in LET-TRF mice^27^. In addition to the attenuation of body weight gain, the parametrial fat tissue weight and inflammation of adipose tissues, all of which are associated with insulin sensitivity, were lower in LET-TRF mice than in LET or CON mice on either the CHOW diet or HFD. These findings suggest that TRF affects body composition by reducing adiposity and adipose tissue inflammation. The restoration of the metabolic profiles might be associated to that of reproductive phenotypes, particularly to changes in hypothalamic-pituitary-ovarian (HPO) axis. In this study, the expression of several key reproductive genes was recovered in LET-TRF mice, without significant body weight changes in the CHOW diet groups. These findings suggest that TRF may have therapeutic effects on the altered HPO axis of the PCOS model rather than only beneficial effects on weight loss and metabolic components. In addition, this study showed that the expression of clock genes, including *Rev-erbα,* in the ovary altered according to LET or TRF treatment. It has been described that rhythmic events in female reproductive physiology are controlled by circadian rhythms^28^. These rhythmic events depend on the coordination of neuroendocrine and endocrine systems, which is facilitated by the timing of gene expression and cellular physiology at each level of the HPO axis^29,30^. Mice with dampened circadian rhythms fed TRF, an imposed daily rhythm in feeding and fasting, maintained a rhythmic expression of circadian-dependent key metabolic regulators, which prevented and reversed metabolic disorders^31–34^. Although, it remains unclear whether circadian rhythms can directly influence the pattern of LH secretion, our findings suggest that the therapeutic effect of TRF could be induced by complex interactions of metabolic, reproductive, or circadian rhythm-associated mechanisms. Further studies are warranted to confirm these findings and evaluate the relevant physiological mechanisms.

To the best of our knowledge, this study is the first to reveal the effects of TRF in an animal model of PCOS. However, some limitations exist. First, ovulatory dysfunction induced by LET does not fully recover after TRF, although menstrual periods were recovered after TRF in the previous clinical study^19^. Further investigations of various TRF regimens are needed to identify a more effective strategy for PCOS. Second, although LET-TRF mice were compared to LET and CON mice, placebo-treated mice subjected to TRF were not investigated here. To fully elucidate the independent effects of LET treatment or TRF, further subdivided studies are needed. Third, investigation for insulin resistance, such as glucose tolerance test, was not conducted in this study. However, both the impaired glucose tolerance in letrozole-treated mice and effect of TRF on glucose tolerance have been described in previous studies^13,25^. Fourth, although several reproductive/clock genes were investigated, the protein levels associated with those genes were not assessed, and the molecular findings do not fully explain the underlying mechanisms of TRF effects and of PCOS phenotype. Further studies on molecular markers associated with PCOS phenotypes and TRF are warranted. Finally, although estrous cycling was investigated in this study, future studies to confirm the effect of TRF on reproductive function in PCOS model should assess the pregnancy rate by obtaining those animals for breeding experiments. Further comparison of characteristics of offspring from those animals is also an interesting topic.

## Conclusions

LET-treated mice showed a phenotype similar to that of women with PCOS, which was partially restored to normal levels after TRF. Thus, TRF could be a potential candidate as an effective dietary regimen for PCOS management. Further studies are warranted to validate these findings and establish a therapeutic treatment for PCOS based on lifestyle modifications.

## Supplementary Information

Supplementary Information.

## Data Availability

The datasets used and/or analyzed during the current study available from the corresponding author on reasonable request.
