# Supplementary material for: Effects of time-restricted feeding on letrozole-induced mouse model of polycystic ovary syndrome
Source: Sci Rep. 2023 Feb 2;13:1943. doi: 10.1038/s41598-023-28260-5 (PMC9894941; doi:10.1038/s41598-023-28260-5)
Supplement: Supplementary file 1 — Supplementary Information. [file 41598_2023_28260_MOESM1_ESM.docx]

Supplementary Table 1. Primers

| Gene | Forward Primer (5`-3`) | Reverse Primer (5`-3`) |
| --- | --- | --- |
| *CYP17a1* | GAGTTTGCCATCCCGAAGGA | CCAGCTCCGAAGGGCAAATA |
| *CYP19a1* | TTTCGCTGAGAGACGTGGAG | AGGATTGCTGCTTCGACCTC |
| *Fshr* | GTGCATTCAACGGAACCCAG | TCTAAGCCATGGTTGGGCAG |
| *Lhr* | GCCATGCATTCAATGGGACG | GGCCTGCAATTTGGTGGAAG |
| *Pgr* | CATACCTTAACTACCTGAGG | GATTAAGCAGATCTTCTGAGG |
| *Amh* | TACTCGGGACACCCGCTATT | CTGATCGATGCTCAGGGTGG |
| *Clock* | ATGGTGTTTACCGTAAGCTGTAG | CTCGCGTTACCAGGAAGCAT |
| *Bmal1* | TGACCCTCATGGAAGGTTAGAA | GGACATTGCATTGCATGTTGG |
| *Per1* | CGGATTGTCTATATTTCGGAGCA | TGGGCAGTCGAGATGGTGTA |
| *Per2* | GAAAGCTGTCACCACCATAGAA | AACTCGCACTTCCTTTTCAGG |
| *Cry1* | CACTGGTTCCGAAAGGGACTC | CTGAAGCAAAAATCGCCACCT |
| *Rev-erbα* | TACATTGGCTCTAGTGGCTCC | CAGTAGGTGATGGTGGGAAGTA |
| *Gnrh* | CTACTGCTGACTGTGTGTTTG | CATCTTCTTCTGCCTGGCTTC |
| *Kiss1r* | GCTCTATCTGCTGCCGCTGC | CTTGAAGCACCAGGAACAGCTGG |
| *GLUT4* | GATCGGCTCTGAAGATGGGG | GGAGGAAATCATGCCACCCA |
